# SP_0916 Is an Arginine Decarboxylase That Catalyzes the Synthesis of Agmatine, Which Is Critical for Capsule Biosynthesis in *Streptococcus pneumoniae*

**DOI:** 10.3389/fmicb.2020.578533

**Published:** 2020-09-18

**Authors:** Moses B. Ayoola, Mary F. Nakamya, Leslie A. Shack, Seongbin Park, Juhyeon Lim, Jung Hwa Lee, Matthew K. Ross, Hyungjin Eoh, Bindu Nanduri

**Affiliations:** ^1^Department of Basic Sciences, College of Veterinary Medicine, Mississippi State University, Starkville, MS, United States; ^2^Zilkha Neurogenetic Institute, University of Southern California, Los Angeles, CA, United States; ^3^Institute for Genomics, Biocomputing and Biotechnology, Mississippi State University, Starkville, MS, United States

**Keywords:** *Streptococcus pneumoniae*, capsular polysaccharide, polyamines, agmatine, metabolomics

## Abstract

The global burden of invasive pneumococcal diseases, including pneumonia and sepsis, caused by *Streptococcus pneumoniae*, a Gram-positive bacterial pathogen, remains a major global health risk. The success of pneumococcus as a pathogen can be attributed to its ability to regulate the synthesis of capsular polysaccharide (CPS) during invasive disease. We previously reported that deletion of a putative lysine decarboxylase (LDC; ΔSP_0916) in pneumococcal serotype 4 (TIGR4) results in reduced CPS. SP_0916 locus is annotated as either an arginine or a LDC in pneumococcal genomes. In this study, by biochemical characterization of the recombinant SP_0916, we determined the substrate specificity of SP_0916 and show that it is an arginine decarboxylase (*speA*/ADC). We also show that deletion of the polyamine transporter (*potABCD*) predicted to import putrescine and spermidine results in reduced CPS, while deletion of spermidine synthase (*speE*) for the conversion of putrescine to spermidine had no impact on the capsule. Targeted metabolomics identified a correlation between reduced levels of agmatine and loss of capsule in Δ*speA* and Δ*potABCD*, while agmatine levels were comparable between the encapsulated TIGR4 and Δ*speE*. Exogenous supplementation of agmatine restored CPS in both Δ*potABCD* and Δ*speA*. These results demonstrate that agmatine is critical for regulating the CPS, a predominant virulence factor in pneumococci.

## Introduction

*Streptococcus pneumoniae* (pneumococcus, Spn) is a Gram-positive bacterium that causes invasive diseases such as pneumonia, meningitis, and sepsis (Schuchat et al., 2001). Pneumococci as pathobionts express virulence factors, including lipoproteins, choline-binding proteins, histidine kinases, and most importantly, capsular polysaccharide (CPS). CPS is a key virulence factor that is the basis for the description of more than 97 serotypes and the target of current vaccines (Gamez et al., 2018). The ability to regulate CPS synthesis is critical for pneumococcal adaptation to different host niches and its success as a commensal and pathogen. Pneumococci can modulate their CPS by a complex phenomenon known as phase variation as well as other independent mechanisms (Manso et al., 2014; Li and Zhang, 2019). Despite decades of research, a comprehensive understanding of CPS regulation in Spn remains elusive.

A single CPS biosynthesis gene cluster is located between the *dexB* and *aliA* genes in the genome of the majority of pneumococcal serotypes (Llull et al., 1999). The first four genes of the CPS operon, *cpsABCD*, are reported to be essential for capsule production (Bender et al., 2003; Morona et al., 2004). However, expression of *cpsA* as a surrogate for the CPS operon is independent of capsule phenotypic change during phase variation (LeMessurier et al., 2006). The role of tyrosine phosphorylation remains contradictory as it has been shown to both enhance and inhibit capsule biosynthesis (Bender et al., 2003; Morona et al., 2004). Competence protein ComE has been reported to be a negative transcriptional regulator of CPS (Zheng et al., 2017). In recent years, the intersection between bacterial physiology and pathogenesis has garnered a lot of attention. For example, expression of pyruvate oxidase (*spxB*) results in reduced CPS during colonization (Overweg et al., 2000), while mutations in *spxB* increase CPS and transcription of *cpsA* (Carvalho et al., 2013a). Deletion of arginine transport in pneumococcal serotype 2 results in a reduced capsule and reduced virulence in an otitis media model of infection (Gupta et al., 2013). On the other hand, impaired arginine transport had no impact on CPS in serotype 4, indicating that these effects could be serotype dependent (Schulz et al., 2014). The precise relationship between arginine metabolism and CPS remains to be established. Uracil, a precursor for pyrimidine biosynthesis, has also been proposed as a regulator of capsule biosynthesis (Carvalho et al., 2013b, 2018). A recent report indicates that different carbon sources in the growth medium impact capsule thickness in pneumococci (Troxler et al., 2019); however, no single metabolite is conclusively linked to the regulation of CPS in pneumococci. We previously reported that altered polyamine metabolism impacts CPS in pneumococci (Nakamya et al., 2018; Ayoola et al., 2019).

Polyamines, such as putrescine, spermidine, cadaverine, and spermine, are ubiquitous, polycationic, aliphatic hydrocarbons that regulate a number of cellular processes (Gevrekci, 2017), and their intracellular concentrations are tightly regulated by transport, biosynthesis, and catabolism (Miller-Fleming et al., 2015). In eukaryotic systems, polyamines are critical for cell proliferation and are being targeted for treatment in certain types of cancers (Casero et al., 2018). While polyamines are reported to be dispensable for growth in some bacteria (Chattopadhyay et al., 2009), they are implicated in pathogenesis. For example, cadaverine reduces the enterotoxin activity of *Shigella flexneri* (Maurelli et al., 1998) and inhibits its phagolytic activity, thereby preventing the spread of this pathogen (Fernandez et al., 2001). Polyamines have also been shown to be essential for biofilm formation, a critical step in the pathogenesis of *Yersinia pestis* (Patel et al., 2006).

Polyamine biosynthesis and transport genes are well conserved in most prokaryotes, including multiple pneumococcal serotypes (Shah et al., 2011). Our previous work indicates that impaired polyamine biosynthesis by the deletion of a putative lysine decarboxylase (LDC; ΔSP_0916), spermidine synthase (∆*speE*), and the polyamine transporter (∆*potABCD*) in pneumococci did not affect *in vitro* growth but resulted in an attenuated phenotype *in vivo* (Shah et al., 2011; Nakamya et al., 2018). We recently demonstrated that deletion of SP_0916 results in reduced capsule and metabolic reprogramming that alters the carbon flux and could limit the availability of precursors for CPS synthesis (Nakamya et al., 2018; Ayoola et al., 2019). Despite its relevance to pneumococcal virulence, predicted function of the enzyme encoded by SP_0916 is inconsistent. SP_0916 is predicted to encode an arginine decarboxylase (ADC) or a LDC. The current annotation in TIGR4 is that of a LDC. The locus corresponding to SP_0916 in D39 is annotated to be an ADC, involved in spermidine synthesis (through indirect evidence), and is essential for the onset of autolysis (Potter and Paton, 2014). Pyridoxal-dependent decarboxylases in the polyamine biosynthesis pathways, such as SP_0916, belong to a family of enzymes with broad substrate specificity that can utilize arginine/lysine/ornithine. Therefore, definitive biochemical data is needed to determine the substrate specificity of SP_0916.

In this study, we determined the substrate specificity of SP_0916 for definitive annotation of this gene. We also evaluated the impact of the deletion of ∆*potABCD* and ∆*speE* on CPS, which are also reported to be attenuated *in vivo*. We measured intracellular concentrations of polyamines and precursors of polyamine synthesis in ∆SP_0916, ∆*potABCD*, and ∆*speE* and correlated these with the capsular phenotype. Our results show that SP_0916 is an ADC. Impaired polyamine transport in *S. pneumoniae* TIGR4 results in reduced CPS, while deletion of SpeE had no impact. Reduced intracellular concentration of agmatine, an intermediate in the putrescine/spermidine synthesis pathway, correlates with reduced CPS expression in ∆*speA* and ∆*potABCD*. Exogenous supplementation of agmatine restores capsule in Δ*speA* and Δ*potABCD*. In summary, we demonstrate that SP_0916, an important virulence gene from polyamine biosynthesis pathway, is an ADC, and that the reaction product agmatine is critical for capsule biosynthesis in pneumococci. Future studies focused on the identification of specific mechanisms by which agmatine regulates CPS are warranted to decipher the polyamine-CPS regulatory network in bacteria.

## Materials and Methods

### Bacterial Strains and Growth Conditions

*Streptococcus pneumoniae* serotype 4 strain (TIGR4; Tettelin et al., 2001) and polyamine synthesis- (ΔSP_0916 and Δ*speE*) and transport- (Δ*potABCD*) deficient strains (Shah et al., 2011; Rai et al., 2016; Nakamya et al., 2018) were used in this study. Bacteria were grown in Todd-Hewitt broth supplemented with 0.5% yeast extract (THY), a rich medium with polyamines to mimic growth *in vivo* or on 5% sheep blood agar plates (BAP) in 5% CO_2_. An isogenic deletion strain of TIGR4 deficient in *speE* was generated by PCR-ligation mutagenesis as described previously. Primers were designed (Table 1) to amplify genomic segments upstream and downstream of *speE* from TIGR4 chromosomal DNA, which were joined by gene splicing, and insertion of the chloramphenicol resistance gene (*cat*) amplified from pIMAY (Monk et al., 2012) by overlap extension (SOEing) PCR (Rai et al., 2016; Thornton, 2016; Nakamya et al., 2018). The recombinant product was transformed into TIGR4 as described previously (Bricker and Camilli, 1999). Transformants were selected on BAP with chloramphenicol (10 μg/ml) and *speE* gene deletion was confirmed by sequencing. Complement strains of Δ*potABCD* and Δ*speE* were generated by cloning *potABCD* and *speE* genes amplified from the TIGR4 genome into the pABG5 vector and transformation of appropriate deletion strains. Transformants were selected on BAP with kanamycin (50 μg/ml) and confirmed by PCR.

**Table 1:** List of primers used in this study.

Primer	Sequence ^*^(5'→3')	Experiment
*speE* upstream FP	GAGCACGGCAAAAAGCCC	Mutagenesis
*speE* upstream RP	CTGCCAAAGCATAATGGGATCCTCCAATCGCTTGACGATTTCCG	Mutagenesis
*cat* FP	ATCCCATTATGCTTTGGCAG	Mutagenesis
*cat* RP	TTATAAAAGCCAGTCATTAGGCC	Mutagenesis
*speE* downstream FP	GGCCTAATGACTGGCTTTTATAAATGTTGCCCAAGTATGTTGAGGAC	Mutagenesis
*speE* downstream RP	TTAGTCCACCATTTGTGGATTTTCA	Mutagenesis
SP_0916-F BamHI	AATTGGATCCAAAGAGTTAGATCAAAACCAAGCCCCAATTTATG	Expression
SP_0916-R XhoI	AATTCTCGAGTTGACTTTTCTTATAGTTTGTCTTTCTCTTAATAACGTTG	Expression

* Underlined sequence complementary to *Streptococcus pneumoniae* TIGR4 chromosomal DNA.

### Enzyme Assay

#### Cloning, Expression, and Purification of SP_0916

The SP_0916 locus encodes a protein of 491 amino acids with a predicted molecular weight of ~54 kDa. This gene (SP_0916) was amplified from chromosomal DNA of *S. pneumoniae* TIGR4 by PCR using primers with BamHI and XhoI (Table 1) restriction sites. The PCR product was restriction digested and cloned into the pET-28a (+) vector (MilliporeSigma, Burlington, MA) with 6x His tag at the C-terminus. The resulting recombinant expression vector pET-28a (+):SP_0916 was transformed into the *Escherichia coli* strain BL21(DE3), which was grown in 100 ml of Luria Bertani medium containing 30 μg/ml kanamycin and 3% ethanol at 37°C to an OD_600nm_ of 0.6 and induced with 1.0 mM 1-thio-β-D-galactopyranoside (IPTG). Five hours post induction, cells were harvested by centrifugation at 3,000 × *g* for 20 min at 4°C, and the cell pellet was stored at −20°C until further use. The frozen pellet was thawed on ice, and resuspended in B-PER reagent buffer (Thermo Fisher Scientific, Waltham, MA, United States) at 4 ml/g of pellet, with 2 μl of benzonase nuclease (Sigma-Aldrich, St. Louis, MO), and 10 μl protease inhibitor/ml (Thermo Fisher Scientific, Waltham, MA, United States), and incubated for 15 min at room temperature.

The cell debris was removed by centrifugation at 15,000 × *g* for 5 min and the lysate was loaded onto a HisPur Cobalt Spin Column (Thermo Fisher Scientific, Waltham, MA, United States). After washing with equilibration/wash buffer containing 100 mM imidazole, the bound proteins were eluted with 500 mM imidazole in elution buffer. The purified protein was desalted using Sephadex G-25 PD-10 column (GE Healthcare, Chicago, IL, United States) equilibrated with phosphate-buffered saline (PBS). All purification steps were performed at 25°C. The quality of purification was evaluated by visualizing the protein by sodium dodecyl sulfate polyacrylamide gel electrophoresis (SDS-PAGE) and staining with Coomassie Brilliant Blue R-250 (Bio-Rad, Hercules, CA, United States). Protein estimation was done according to the BCA method using the Pierce BCA Protein Assay kit and following manufacturer’s instructions (Thermo Fisher Scientific, Waltham, MA, United States).

#### Enzyme Kinetics Assay

To evaluate the decarboxylase activity of recombinant SP_0916, the rates of substrate/product conversions of arginine/agmatine, lysine/cadaverine, and ornithine/putrescine were assessed by a liquid chromatography-mass spectrometry (LC-MS) method. The enzyme (final concentration of protein 50 μg/ml) was mixed with increasing concentration of substrate (0.01–10 mM) in 50 mM Tris-HCl buffer (pH 8.0) containing 2.5 mM MgSO_4_ and 0.6 mM pyridoxal 5′-phosphate (PLP). The reactions were incubated for 15 min at 37°C in dark and terminated with 12.5 μl of 70% (w/v) perchloric acid. After sitting on ice for at least 10 min, the samples were neutralized with 25 μl of 10 N KOH and extracted twice with 1 ml of 1-butanol. The pooled organic layer was dried under nitrogen gas and reconstituted with 100 μl of aqueous 10 mM ammonium acetate containing *n*-heptylamine as an internal standard.

Analysis of the extracts was performed on a Surveyor LC-MS system (MSQ, Thermo Fisher Scientific, San Jose, CA, United States). The analytical column used was a Phenomenex Synergi Fusion-RP (150 × 3 mm, 4 μm, 80 Å) set at a column temperature of 30°C. The mobile phases consisted of (A) aqueous 10 mM ammonium acetate, (B) acetonitrile containing with 0.1% v/v formic acid, and (C) aqueous 10 mM ammonium acetate containing with 0.1% v/v formic acid. The gradient program was 0 min (95% A and 5% B), 0.75 min (95% A and 5% B), 5 min (20% B and 80% C), 9 min (20% B and 80% C), 11 min (40% B and 60% C), 15 min (95% A and 5% B), and 22 min (95% A and 5% B). The flow rate was 0.5 ml/min and the column eluate was directed into the mass spectrometer using heated electrospray ionization in positive ion mode. Optimum cone voltage was determined for each analyte by post-column infusion of the individual compounds into a 50% A/50% B blend of mobile phase being pumped at a flow rate 0.5 ml/min. The MSQ conditions were set as follows: probe temperature, 400°C; capillary voltage, 3.5 kV; nitrogen nebulizer pressure, 80 psi. Xcalibur software was employed for data acquisition and processing. For quantification, calibration standards were prepared ranging from 0.01 to 10 mM. Calibration curves were constructed for agmatine, cadaverine, and ornithine. The change in velocity with increase in substrate concentrations was fitted with Sigma Plot v. 12 to estimate the kinetic parameters using the Michaelis-Menten equation by non-linear regression method, and all experiments were carried out with three independent replicates. The kinetic parameters turnover number (*k*_cat_), Michaelis-Menten constant (*K*_m_), and catalytic efficiency (*k*_cat_/*K*_m_) were obtained.

### Targeted Metabolomics

We performed targeted metabolomics to measure polyamines, precursors, and intermediates of polyamine biosynthesis and degradation. Wild type (WT) TIGR4, ΔSP_0916, Δ*potABCD*, and Δ*speE* cells cultured in THY (mid-log phase, *n* = 8) were washed twice in PBS to remove possible polyamine contamination from the growth medium and collected separately onto a Whatman polycarbonate membrane, 0.2 μm by vacuum filtration (Thermo Scientific, Rockford, IL). The membranes were flash-frozen in liquid nitrogen and stored at −80°C until further use. Polyamines and other metabolites were extracted from the cells on the membranes with 40:40:20 (v/v/v) acetonitrile:methanol:water, and 100 mM formic acid by incubation at −20°C for 15 min and subsequent centrifugation (16,000 × *g*, 5 min, 4°C). The supernatant was filtered through a Spin-X column (16,000 × *g*, 10 min, 4°C) prior to mass spectrometry. Metabolite differentiation and detection were performed using published protocols (Eoh and Rhee, 2013; Eoh et al., 2017). LC-MS–based metabolomics analysis was performed with an Agilent Accurate Mass 6230 TOF coupled with an Agilent 1290 LC system using a Cogent Diamond Hydride Type C column. Briefly, the mobile phase consisted of the following: solvent A (ddH_2_O with 0.2% formic acid) and solvent B (acetonitrile with 0.2% formic acid). The gradient used was as follows: 0–2 min, 85% B; 3–5 min, 80% B; 6–7 min, 75% B; 8–9 min, 70% B; 10–11.1 min, 50% B; 11.1–14 min 20% B; and 14.1–24 min 5% B followed by a 10 min re-equilibration period at 85% B at a flow rate of 0.4 ml/min. Mass axis dynamics was calibrated by continuous infusion of a reference mass solution using an isocratic pump. This configuration achieved mass errors of 5 ppm, mass resolution ranging from 10,000 to 25,000 (over *m*/*z* 121–955 atomic mass units), and 5 × log10 dynamic range. Metabolite identities were searched for using a mass tolerance of <0.005 Da. Metabolite concentrations from the eight biological replicates were normalized to biomass based on measurement of residual peptide content in individual samples using the Pierce BCA Protein Assay kit (Thermo Scientific, Rockford, IL). Data were analyzed using Profinder B.07.00 software (Agilent Technologies, Santa Clara, CA). Extracted molecular features detected in the mass analyzer were identified using accurate mass values and used to generate empirical molecular formulae using an in-house metabolite database that included known intermediates, amino acid precursors, and polyamines and their derivatives. Statistical analysis of metabolite peak intensity data was performed using MetaboAnalyst 4.0 (Chong et al., 2018). Data were normalized (quantile), log transformed, and significant fold change between WT and deletion strains were identified by Student’s *t*-test (*p* ≤ 0.05).

### Immunoblot Estimation of Pneumococcal Total Capsular Polysaccharide

Total CPS was quantified by immunoblot assays as described earlier (Nakamya et al., 2018). An isogenic capsular variant of TIGR4 (T4R), in which the CPS locus is replaced with the Janus cassette resulting in an unencapsulated phenotype (Fernebro et al., 2004) was used as a negative control. Bacteria were cultured in THY supplemented with 10% fetal bovine serum with or without agmatine (20 mM) to an OD_600nm_ of 0.2. An aliquot of bacterial culture was plated on BAP for colony forming unit (CFU) enumeration and 1 ml bacteria was stored at 2–8°C until further use. The CFUs for all strains were ~9.0 × 10^7^/ml. CPS was extracted in a lysis buffer (4% deoxycholate, 50 μg/ml DNAse I, and 50 μg/ml RNAse A) at 37°C for 10 min and centrifuged at 18,000 × *g* for 10 min. Supernatant (2 μl) was spotted on a 0.2-μm-pore-size nitrocellulose membrane (Thermo Fisher Scientific, Waltham, MA, United States) and oven dried at 60°C for 15 min. The membranes were blocked and incubated with either a mouse monoclonal antibody, a gift from Moon H. Nahm (Birmingham, AL, United States), or a rabbit anti-serotype 4 polyclonal antibody (Cedarlane, Burlington, NC, United States) at 1:1000. Secondary antibody for the monoclonal was horseradish peroxidase (HRP)-conjugated goat anti-mouse antibody and for the polyclonal was HRP-conjugated goat anti-rabbit antibody (Thermo Fisher Scientific, Waltham, MA, United States) at 1:10,000. Membranes were developed with enhanced chemiluminescence (ECL) detection (Thermo Fisher Scientific, Waltham, MA, United States) and scanned using a ChemiDoc XRS+ with Image Lab software (Bio-Rad, Hercules, CA, United States).

### Estimation of Surface Exposed Phosphocholine

Comparison of surface exposed phosphocholine (PC) levels between the WT and polyamine metabolism deletion strains was performed as described previously (Ayoola et al., 2019). Briefly, 300 μl of mid-exponential-growth-phase bacteria were pelleted and washed in 1X PBS. Pellets were resuspended in 100 μl of unconjugated IgA, Kappa from murine myeloma anti-phosphocholine (Sigma-Aldrich, St. Louis, MO) at 1:100 in 1X PBS and incubated on ice for 30 min. The binding reaction was stopped with 500 μl of 1X PBS and centrifuged at 4,000 × *g* for 5 min. Pellets were resuspended in 100 μl of phycoerythrin (PE)-conjugated rat anti-mouse IgA secondary antibody (Thermo Fisher Scientific, Waltham, MA) at 1:100 in 1X PBS and incubated at 4°C, in the dark, for 30 min. Staining reactions were stopped with 500 μl PBS, and products were pelleted and re-suspended in 300 μl of 2% paraformaldehyde. Samples (10,000 events) were collected, analyzed, and plotted using an Attune Acoustic Focusing Cytometer (Life Technology, Foster City, CA, United States).

## Results

### SP_0916 Encodes an Arginine Decarboxylase

**Figure 1:**
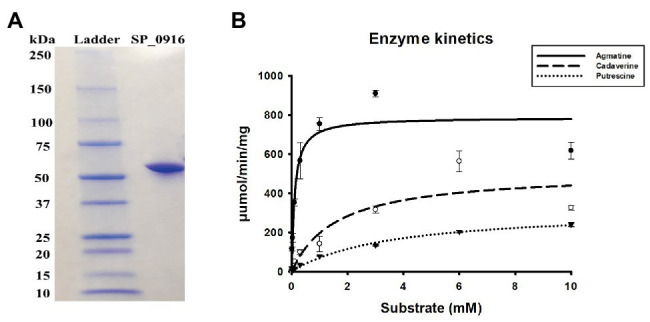
Gel electrophoresis and enzyme kinetics of SP_0916. **(A)** Overexpressed and purified recombinant SP_0916 was resolved by sodium dodecyl sulfate polyacrylamide gel electrophoresis (SDS-PAGE) and stained with Coomassie Brilliant Blue R-250 for visualization of the 54 KDa protein. The ladder lane on the left is the protein marker showing the molecular mass standards. **(B)** Enzyme kinetics for the conversion of arginine to agmatine, lysine to cadaverine, and ornithine to putrescine were performed in triplicate using liquid chromatography-mass spectrometry (LC-MS). The change in velocity with increase in substrate concentrations was fitted with Sigma Plot v.12 to estimate the kinetic parameters using the Michaelis-Menten equation by non-linear regression method. The *K*_m_ of SP_0916 with the substrates is ornithine (3.55 ± 0.28 mM) > lysine (1.61 ± 0.28 mM) > arginine (0.11 ± 0.02 mM), indicating that arginine is the preferred substrate of this decarboxylase. High catalytic efficiency (*k*_cat_/*K*_m_) for the SP_0916-catalyzed conversion of arginine to agmatine (4.0 × 10^5^ min^−1^mM^−1^) compared to low *k*_cat_/*K*_m_ for conversion of lysine to cadaverine (1.7 × 10^4^ min^−1^mM^−1^) and ornithine to putrescine (4.8 × 10^3^ min^−1^mM^−1^) show that arginine is the preferred substrate for SP_0916. *k*_cat_/*K*_m_ values are obtained using the means for each kinetic parameter.

Current genome annotation of *S. pneumoniae* TIGR4 in NCBI (Coordinators, 2016), KEGG (Kanehisa, 2019), and STRING (Szklarczyk et al., 2019) databases indicate that SP_0916 encodes a putative LDC that catalyzes the synthesis of cadaverine in several organisms, including humans. However, annotation in BioCyc database (Karp et al., 2019) and the work of Potter and Paton (2014) indicate that SP_0916 encodes an ADC that catalyzes the conversion of arginine to agmatine in the putrescine/spermidine biosynthesis pathway. We determined the substrate specificity of SP_0916. Recombinant SP_0916 containing a C-terminus 6× His tag was overexpressed in the *E. coli*. SDS-PAGE analysis of purified SP_0916 protein indicated a 54 kDa protein (Figure 1A). Using LC-MS to measure reaction end products, the kinetic parameters of recombinant SP_0916 were determined with the substrates arginine, lysine, and ornithine (Figure 1B). Estimation of kinetic parameters using Michaelis-Menten equation shows that SP_0916 is an SpeA. The Michaelis-Menten constant (*K*_m_) of SP_0916 with the substrates is ornithine (3.55 ± 0.28 mM) > lysine (1.61 ± 0.28 mM) > arginine (0.11 ± 0.02 mM), indicating that arginine is the preferred substrate of this decarboxylase; goodness of fit (*R*^2^) to the Michaelis-Menten model was 0.96, 0.91, and 0.99 for arginine, lysine, and ornithine, respectively. The catalytic efficiency (*k*_cat_/*K*_m_) of SP_0916 for the conversion of arginine to agmatine (4.0 × 10^5^ min^−1^mM^−1^) is ~24-fold greater than that for the conversion of lysine to cadaverine (1.7 × 10^4^ min^−1^mM^−1^) and ~83-fold greater than the conversion of ornithine to putrescine (4.8 × 10^3^ min^−1^mM^−1^). These results demonstrate that SP_0916 is an arginine decarboxylase and it will be referred to as ADC in the following sections.

### Impaired Polyamine Transport Results in Reduced CPS

**Figure 2:**
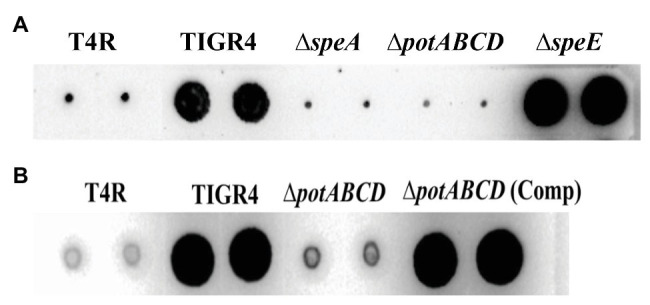
Immunoblot analysis of total capsular polysaccharide (CPS) in WT, polyamine synthesis/transport deletion and complemented strains. All strains were cultured to mid-log phase and total CPS isolated from equal number of cells for each strain. Representative immunoblots using serotype 4 specific antibody from three independent experiments are shown. **(A)** CPS from unencapsulated T4R, TIGR4, Δ*speA* (Nakamya et al., 2018), polyamine transport deficient Δ*potABCD* and Δ*speE*. **(B)** CPS from T4R, TIGR4, Δ*potABCD*, and Δ*potABCD* complement.

To determine the impact of the deletion of Δ*speE* and the polyamine transporter Δ*potABCD* on CPS, if any, we estimated total CPS of deletion strains and WT by immunoblot assays using a monoclonal anti-serotype 4 antibody. Deletion of polyamine transport resulted in a reduced capsule in Δ*potABCD* that is comparable to the levels in T4R, an isogenic variant of TIGR4 that is unencapsulated (Figure 2A), and Δ*speA* (Nakamya et al., 2018). On the other hand, total CPS from Δ*speE* is comparable to that of WT TIGR4 (Figure 2A). Complementation of Δ*potABCD* with a pABG5-*potABCD* construct [Δ*potABCD* (Comp)] fully restored CPS to the levels comparable to that of WT (Figure 2B). Total CPS from Δ*speE* and Δ*speE* complement strain (pABG5-*speE*) were comparable, while culture supernatants for all bacterial strains had no detectable CPS (data not shown). These results clearly demonstrate that deletion of the polyamine transporter operon *potABCD* in *S. pneumoniae* results in reduced CPS.

**Figure 3:**
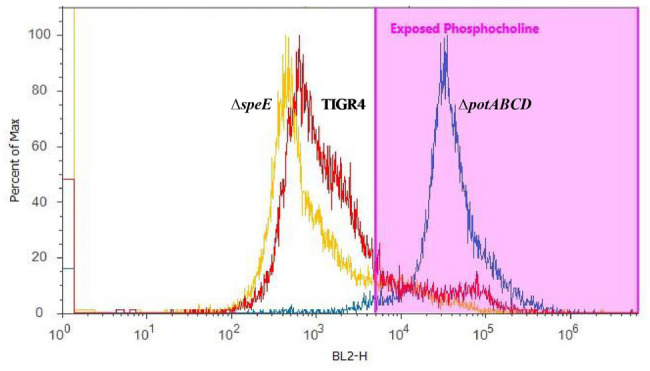
Measurement of surface-exposed phosphocholine (PC) in TIGR4 and deletion strains. Pneumococci were grown to exponential phase and PC was measured by flow cytometry. PC was stained with an unconjugated Kappa murine myeloma IgA anti-phosphocholine antibody, followed by detection with a phycoerythrin (PE)-conjugated rat anti-mouse secondary antibody. Samples were fixed in 2% paraformaldehyde and histogram heights read in blue laser 2 channel (BL2-H) on an Attune Acoustic Focusing Cytometer. The gate was set based on a negative control that was treated with secondary antibody only. Representative histogram overlay plots, from three independent experiments, of the fluorescence intensity of murine myeloma IgA antibody binding to exposed PC on TIGR4 (red), Δ*speE* (yellow), and Δ*potABCD* (blue) are shown.

To corroborate changes in the capsule phenotype detected by the immunoblot assay, we measured surface exposed PC by flow cytometry. Loss of capsule is expected to render the cell surface more permeable to PC antibody, thereby increasing the detection of PC on the cell surface. PC was more readily accessible in Δ*potABCD* compared to Δ*speE* and TIGR4, confirming reduced levels of CPS in Δ*potABCD* strain (Figure 3).

### Intracellular Concentrations of Polyamines and Precursors/Intermediates of Polyamine Synthesis Pathways

**Figure 4:**
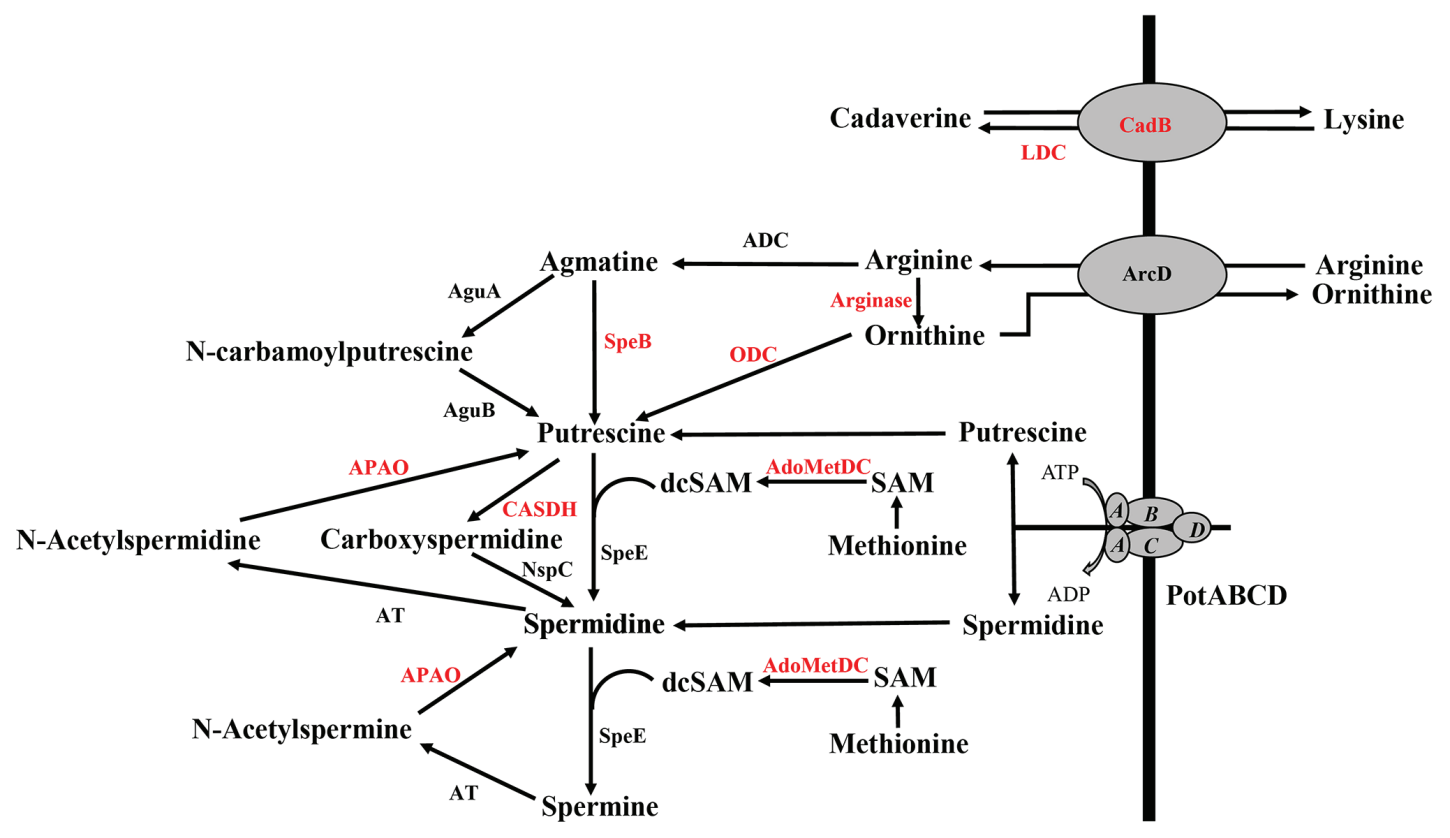
Polyamine transport and synthesis pathways. Cadaverine is synthesized by decarboxylation of lysine by putative lysine decarboxylase (LDC), while CadB serves as an antiporter for cadaverine and lysine exchange. Arginine decarboxylation by arginine decarboxylase (ADC) generates agmatine, a precursor for putrescine. Enzymatic activities of agmatine deiminase (AguA) and carbon-nitrogen hydrolase family protein (AguB) convert agmatine to putrescine in two steps or agmatine directly to putrescine by agmatinase (SpeB). The conversion of ornithine directly to putrescine by ornithine decarboxylase (ODC) is considered to be the main evolutionary pathway of polyamine biosynthesis in most organisms, but this gene is not currently annotated in pneumococcal genomes (shown in red). Ornithine/arginine antiporter (ArcD) regulates intracellular concentrations of arginine and ornithine. Putrescine can be converted to spermidine and spermine in sequential steps using decarboxylated S-adenosylmethionine [dcSAM, catalyzed by adenosylmethionine decarboxylase (AdoMetDC)] from methionine as a methyl donor by the enzymatic activity of spermidine synthase (SpeE). Alternatively, putrescine can be converted to carboxyspermidine and spermidine by carboxyspermidine dehydrogenase (CASDH) and carboxyspermidine decarboxylase (NspC), respectively. Pneumococcal genomes encode a single polyamine transporter (PotABCD) that is predicted to import spermidine and putrescine. Polyamine catabolism includes acetylation and thereby sequestration by a polyamine acetyltransferase (AT). Polyamine oxidase (APAO) that catalyzes the reverse reactions, i.e., generates free polyamines from acetylated forms (and other enzymes shown in red), are not annotated in the genome, at present.

Deletion of polyamine transport and synthesis is expected to alter intracellular concentrations of polyamines such as putrescine and spermidine. We measured polyamines, amino acid precursors, and intermediates of polyamine metabolism (Figure 4) in polyamine biosynthesis (Δ*speA* and Δ*speE*) and transport (Δ*potABCD*) deletion strains and the WT strain using LC-MS. In addition to arginine, lysine, and ornithine, we also measured the intermediates agmatine, methionine, precursor for co-factor decarboxylated *S*-adenosylmethionine, and *N*-acetylspermine and *N*-acetylspermidine, which are degradative products of spermine and spermidine, respectively (Figure 4). Our results show that intracellular concentration of agmatine is significantly reduced in Δ*speA* and Δ*potABCD*, which have reduced CPS (Table 2). Agmatine is the product of arginine decarboxylation catalyzed by ADC (Figure 4). PotABCD transporter is predicted to import spermidine and putrescine in pneumococci, and our results show a significant reduction of these two polyamines in Δ*potABCD* (Table 2). Compared to WT, only *N*-acetylspermine is higher in Δ*potABCD*. Although biosynthesis does not appear to compensate for the loss of transport, intact polyamine transport in Δ*speA* and Δ*speE* helps maintain spermidine and putrescine levels (Table 2). Agmatine is among the most affected metabolites in Δ*potABCD* with ~59-fold reduction in intracellular concentration (Table 2). Similar to total CPS being comparable between WT and Δ*speE* (Figure 2), all measured metabolites, including agmatine, are comparable between these two strains (Table 2), indicating that maintaining agmatine levels is essential for CPS synthesis in Spn.

**Table 2:** Significant changes in the levels of metabolites from polyamine synthesis pathways in synthesis and transport impaired pneumococci relative to the wild type (WT) strain.

Compounds	Δ*speA*/TIGR4	Δ*potABCD*/TIGR4
Agmatine	−21.1	−58.6
Arginine	n.s	−1.9
Cadaverine	n.s	n.s
Lysine	n.s	−1.9
Methionine	1.7	n.s
*N*-acetylspermidine	1.5	−8.2
*N*-acetylspermine	2.1	1.9
Ornithine	n.s	−1.6
Putrescine	n.s	−2.2
*S*-adenosylmethionine	n.s	−1.7
Spermidine	1.7	−58.7
Spermine	n.s	n.s

n.s = no significant difference at *p* ≤ 0.05. The magnitude of change in the ratio and the direction of change (i.e., positive for increase and negative for decrease) are shown. None of the metabolite concentrations in Δ*speE* were found to be significantly different than those in strain TIGR4.

### Agmatine Is Critical for Capsule Biosynthesis

**Figure 5:**
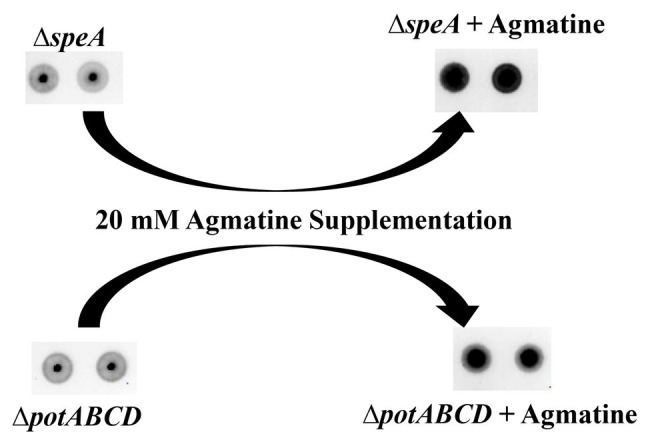
Agmatine is critical for capsule biosynthesis in pneumococcus. Total CPS isolated from Δ*speA* and Δ*potABCD* cultured in THY or THY supplemented with agmatine [20 mM of agmatine, a quarter of minimum inhibitory concentration (MIC)] was spotted on a nitrocellulose membrane. Probing with anti-serotype 4 specific antibody and a horseradish peroxidase (HRP)-conjugated secondary antibody, the membrane was developed with enhanced chemiluminescence (ECL) detection and scanned using a ChemiDoc XRS+ with Image Lab software (Bio-Rad, Hercules, CA, United States). Agmatine successfully restored capsule in the two unencapsulated Δ*speA* and Δ*potABCD* strains in three independent experiments.

Deletion of the (Δ*potABCD* and Δ*speA*; Nakamya et al., 2018) results in loss of CPS, while deletion of spermidine synthase (Δ*speE*) has no impact on the capsule (Figure 2). Measurement of intracellular polyamines, precursors of synthesis and intermediates, suggests a correspondence between reduced intracellular agmatine and reduced CPS. To determine whether agmatine is critical for CPS production, we conducted agmatine supplementation assays with Δ*speA* and Δ*potABCD* and estimated total CPS. The minimum inhibitory concentration (MIC) of agmatine is 80 mM (data not shown). Agmatine supplementation vs. CPS restoration by immunoblot assay dose response measured at 5, 10, 20, and 40 mM agmatine indicated that one quarter MIC (20 mM) restores CPS in ∆*speA* (Supplementary Figure S1). Therefore, we carried out supplementation assays with 20 mM agmatine. Our results show that one quarter MIC agmatine restores the encapsulated phenotype in Δ*potABCD* and Δ*speA* (Figure 5). Supplementation of an equivalent MIC (one quarter MIC) of putrescine (0.57 mM) and spermidine (0.43 mM) failed to restore capsule in the Δ*speA* strain that has an intact potABCD for the import of these polyamines (data not shown). However, it must be noted that supplemented levels of agmatine (i.e., 5 mM) that were approximately 10 times the amount of supplemented putrescine or spermidine also failed to restore capsule in the Δ*speA* strain.

## Discussion

Modulation of polyamine synthesis and transport is being targeted for antiproliferative therapy, given the absolute requirement of polyamines for growth of mammalian cells (Casero et al., 2018). Recent studies demonstrate that polyamine metabolism is at the interface of host-pathogen interactions during pathogenesis. Signature tagged mutagenesis studies indicated that pneumococcal polyamine transport (*potD*) and synthesis (SP_0916) are essential for pathogenesis (Polissi et al., 1998; Hava and Camilli, 2002). Murine models of nasopharyngeal colonization and invasive disease confirm that polyamine synthesis and transport genes are essential for virulence of pneumococci (Shah et al., 2011). Immunization studies with PotD protein, either alone (Shah and Swiatlo, 2006; Shah et al., 2009) or in combination with other pneumococcal proteins (Min et al., 2012; Converso et al., 2017), clearly establish that polyamine transport protein is a potent immunogen that affords protection against colonization and invasive infections. Characterization of Spn TIGR4 ΔSP_0916 showed that modulation of polyamine homeostasis by deletion of this synthesis gene results in reduced capsule (Nakamya et al., 2018), which could explain the reported attenuation *in vivo* (Shah et al., 2011). Although there is evidence for a strong link between polyamine metabolism and pneumococcal virulence, specifically on CPS, there are many gaps in our knowledge.

Description of polyamine synthesis pathways in pneumococci is based on what is known in *E. coli*. Polyamine synthesis occurs *via* decarboxylation of amino acids such as arginine, ornithine, and lysine in multiple pathways (Figure 4). Putrescine synthesis from ornithine in a single step reaction catalyzed by ornithine decarboxylase (ODC) is the predominant pathway in eukaryotic systems and is not annotated in Spn. However, additional biosynthesis pathways exist that convert arginine to putrescine in two consecutive enzymatic steps catalyzed by ADC and SpeB. The current annotation of SP_0916 in TIGR4, the focus of this study, is inconsistent; it is described as either an ADC or a LDC (*cadA*, synthesizes cadaverine from lysine) in pneumococcal genomes. For example, the locus corresponding to SP_0916 in D39 is ADC, involved in spermidine synthesis (Potter and Paton, 2014). This annotation is based on indirect experimental evidence, not by direct measurement of kinetics with different substrates. In the absence of definitive experimental evidence, we used the status quo at the time of publication (i.e., *cadA*) in our earlier work. In this study, we undertook biochemical characterization of SP_0916, a well-documented virulence gene in pneumococci (Hava and Camilli, 2002; Shah et al., 2011; Nakamya et al., 2018). The biochemical characterization of recombinant SP_0916 reported here clearly demonstrates that SP_0916 is indeed an ADC, although it can utilize lysine and ornithine substrates with significantly lower catalytic efficiency. However, the *K*_m_ of SP_0916 with arginine (~100 μM) is 3-fold higher than that of the *E. coli* ortholog (30 μM; Wu and Morris, 1973). While the data presented here annotates the enzymatic function of SP_0916, it also illustrates the gaps in the annotation of pneumococcal genomes, which now appear to lack a LDC. The pyridoxal-dependent decarboxylase family of enzymes that catalyze the synthesis of polyamines are predicted to utilize multiple substrates. Additional pyridoxal-dependent decarboxylases in the TIGR4 genome include SP_0166. Characterization of SP_0166 will help annotate polyamine synthesis pathways in Spn TIGR4.

Using capillary electrophoresis, we previously reported that intracellular levels of spermidine, putrescine, and cadaverine were reduced in Δ*speA*, Δ*potABCD*, and Δ*speE* compared to WT TIGR4 strain. However, with this method, the differences we reported were qualitative, as we could not attribute any significance to the identified differences (Shah et al., 2011). Using a relatively more sensitive LC-MS approach, in this study, we measured intracellular concentrations of polyamines and precursors in polyamine metabolism impaired pneumococci. The metabolic profile of Δ*speA* confirms that it encodes an arginine decarboxylase, as agmatine levels are significantly depleted in this strain, while cadaverine levels were comparable to that of the WT. Transport plays a significant role in meeting cellular needs for polyamines and maintaining polyamine homeostasis. The single putative polyamine transport operon, *potABCD*, annotated in pneumococcal genomes, is predicted to transport both putrescine and spermidine from the extracellular matrix. The metabolic profile of Δ*potABCD* confirms that PotABCD is indeed a transporter that imports both spermidine and putrescine. Studies are underway to determine the substrate specificity of PotABCD. Characterization of pneumococcal surface in Δ*potABCD* and Δ*speE* indicates that loss of polyamine transport results in reduced CPS, while deletion of spermidine synthesis has no impact on CPS. Since the WT and deletion strains were cultured in a complete medium that provides polyamines, there is no additional nutritional stress on the cells. Use of a rich medium that contains polyamines mimics host microenvironments that have polyamines and allows one to determine the impact of a gene deletion on a phenotype that is relevant *in vivo*, such as the capsule, despite the compensation by transport and additional mechanisms that are yet to be identified. Although Δ*speE* harbors a deletion in spermidine synthesis, it may not constitute the predominant route for spermidine synthesis (Potter and Paton, 2014), as the enzyme required to generate synthesis of decarboxylated *S*-adenosylmethionine (Figure 4) is not annotated in pneumococcal genomes at present. Nevertheless, *speE* has been shown to be important for spermidine synthesis. Furthermore, transport could compensate for spermidine synthesis in Δ*speE* (Table 2). Thus, spermidine synthesis catalyzed by SpeE does not appear to be critical for CPS synthesis in Spn. Therefore, the reported attenuation of this strain in murine models could involve capsule-independent mechanisms. Characterization of Spn with genetic deletions in spermidine synthesis *via* carboxyspermidine will help determine the relative importance of this pathway in spermidine synthesis and possibly in CPS regulation.

Agmatine levels were significantly reduced in both Δ*speA* and Δ*potABCD* (Table 2), which had reduced capsule, and were comparable between WT and Δ*speE*, which were encapsulated. Exogenous supplementation with agmatine restores CPS, suggesting that agmatine is critical for CPS biosynthesis. The concentration of agmatine used for supplementation is neither a relevant pharmacological nor physiological dose. However, it is useful as a tool to determine whether agmatine is necessary for CPS synthesis. Relatively high MIC for agmatine compared to other polyamines such as putrescine and spermidine indicates inefficient transport of agmatine in Spn. Although there is an arginine-agmatine antiporter in *E. coli*, agmatine uptake systems in Spn are yet to be identified and characterized. Current annotation of Spn TIGR4 genome has SP_1001, a locus that encodes an amino acid permease family protein with the potential to transport amino acids, polyamines, and agmatine. Future studies to determine the substrate specificity of SP_1001 are warranted.

Reduced levels of agmatine in Δ*potABCD* could be due to significant reduction in the intracellular concentration of arginine, the precursor for this intermediate in polyamine biosynthesis (Table 2). Pneumococcus is an arginine auxotroph that largely depends on an extracellular source of this essential amino acid and the genome encodes ArcD, an arginine-ornithine antiporter. Deletion of *arcD* in serotype 2 has been reported to inhibit pneumococcal capsule synthesis by an unknown mechanism and virulence during colonization and establishment of otitis media infection (Gupta et al., 2013). This reported loss of CPS in Δ*arcD* could involve adverse effects on spermidine/putrescine synthesis, specifically agmatine synthesis from arginine. It is likely that Δ*potABCD* has to ration the available arginine between polyamine biosynthesis and other competing pathways that utilize this amino acid. Reduced arginine in Δ*potABCD* could contribute to reduced levels of putrescine and spermidine by biosynthesis, apart from the direct impact due to loss of import of these two polyamines. We previously reported the importance of pneumococcal polyamine transport in inhibiting the host response (Rai et al., 2016). Recombinant PotD protein affords protection against invasive pneumococcal disease in murine models (Shah and Swiatlo, 2006; Shah et al., 2009; Converso et al., 2017). Studies with unencapsulated strains show that polyamine transport is not required for colonization or infection of the host tissues (Pipkins et al., 2017), although equivalent information on the contribution of polaymine synthesis remains unexplored.

Agmatine and its role in eukaryotic systems (Piletz et al., 2013) is well documented, with the neuroprotective role and the ability to trigger the innate immune response being the most studied (Paulson et al., 2014; Kotagale et al., 2019). There is evidence to suggest that agmatine reduces glycolysis, increases gluconeogenesis and fatty acid oxidation, and ultimately causes weight reduction in rats (Nissim et al., 2014). In *Pseudomonas aeruginosa*, agmatine regulates biofilm formation (Williams et al., 2010) and inhibits inflammatory response by host immune cells (McCurtain et al., 2019). A recent report demonstrates the essential role of *E. coli*-derived agmatine in regulating metabolism, specifically fatty acid metabolism *via* interaction with metformin in *Caenorhabditis elegans* (Pryor et al., 2019). Therefore, it is likely that in pneumococci, agmatine regulates CPS, probably by modulating glycolysis and/or fatty acid metabolism that impacts availability of precursors for CPS synthesis, as we reported earlier (Ayoola et al., 2019). This metabolic reprogramming could involve regulation by a number of transcription factors, including catabolite control protein A (CCPA) and CodY, which sense the metabolic state and regulate polyamine pathways that ultimately impact CPS.

In conclusion, this study determines the substrate specificity of SP_0916 and demonstrates that it is an ADC that catalyzes the synthesis of agmatine. Agmatine is an intermediate in the putrescine/spermidine biosynthesis pathway and is critical for regulating CPS in pneumoccci. Deletion of polyamine transport has adverse effect on the capsule, a critical virulence factor in pneumococci, that could explain the reported attenuation *in vivo*. Modulation of polyamine homeostasis impacts pneumococcal virulence. A comprehensive description of polyamine metabolic pathways is warranted to leverage this system for developing novel theraputic strategies for treating pneumococci, which poses risk to human health worldwide.

## Data Availability Statement

The original contributions presented in the study are included in the article/Supplementary Material; further inquiries can be directed to the corresponding author.

## Author Contributions

BN conceived, supervised, and designed the experiments. MA performed the experiments and drafted the manuscript. MN, LS, SP, JHL, and JL performed experiments. MR assisted with enzyme kinetics analysis. HE supervised the metabolomics analysis. BN and MA finalized the draft. All authors contributed to the article and approved the submitted version.

### Conflict of Interest

The authors declare that the research was conducted in the absence of any commercial or financial relationships that could be construed as a potential conflict of interest.

## Abbreviations

Spn: *Streptococcus pneumoniae*
CPS: Capsular polysaccharide
LDC: Lysine decarboxylase
*speA*/ADC: Arginine decarboxylase
SpeE: Spermidine synthase
PotABCD: Polyamine transporter
SDS-PAGE: Sodium dodecyl sulfate polyacrylamide gel electrophoresis
*K*_m_: Michaelis-Menten constant
*V*_max_: Maximum velocity
*k*_cat_/*K*_m_: Catalytic efficiency
