# Supplementary material for: SP_0916 Is an Arginine Decarboxylase That Catalyzes the Synthesis of Agmatine, Which Is Critical for Capsule Biosynthesis in Streptococcus pneumoniae
Source: Front Microbiol. 2020 Sep 18;11:578533. doi: 10.3389/fmicb.2020.578533 (PMC7531197; doi:10.3389/fmicb.2020.578533)
Supplement: Supplementary file 1 [file Data_Sheet_1.docx]

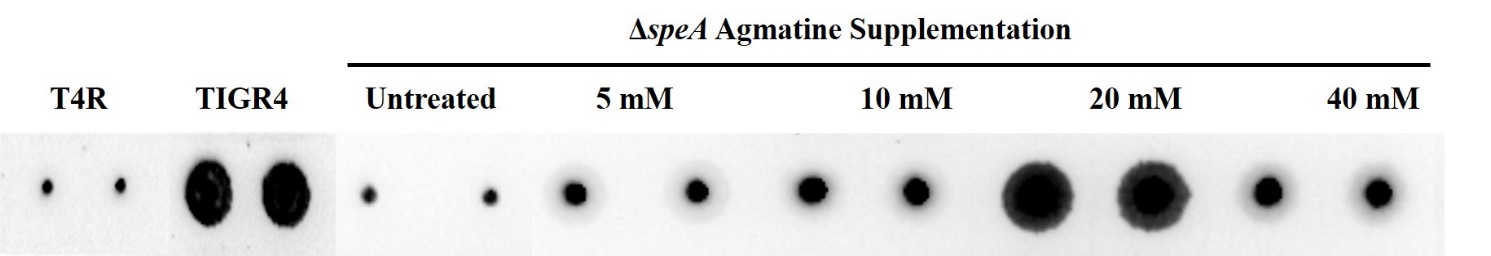


**Supplementary Figure S1.** Exogenous supplementation of agmatine restores capsule in arginine decarboxylase deficient pneumococci. Total CPS isolated from unencapsulated T4R, encapsulated TIGR4, Δ*speA* cultured in THY or THY supplemented with 5, 10, 20 and 40 mM of agmatine was normalized to CFU and spotted on a nitrocellulose membrane. Probing with anti-serotype 4 specific antibody and a horseradish peroxidase (HRP)-conjugated secondary antibody, the membrane was developed with enhanced chemiluminescence (ECL) detection and scanned using a ChemiDoc XRS+ with Image Lab software (Bio-Rad, Hercules, CA, USA). While there is partial restoration of CPS at 5 and 10 mM agmatine, 20 mM agmatine fully restores CPS in Δ*speA*.
